# Atmospheric Degradation of Ecologically Important Biogenic Volatiles: Investigating the Ozonolysis of (*E*)-β-Ocimene, Isomers of α and β-Farnesene, α-Terpinene and 6-Methyl-5-Hepten-2-One, and Their Gas-Phase Products

**DOI:** 10.1007/s10886-023-01467-6

**Published:** 2024-01-09

**Authors:** Dalila Touhami, Adedayo O. Mofikoya, Robbie D. Girling, Ben Langford, Pawel K. Misztal, Christian Pfrang

**Affiliations:** 1 Department of Chemistry, School of Chemistry, Food and Pharmacy, University of Reading, Whiteknights, Reading, RG6 6DX UK; 2 School of Agriculture, Policy and Development, University of Reading, Whiteknights, Earley Gate, Reading, RG6 6EU UK; 3 Centre for Sustainable Agricultural Systems, Institute for Life Sciences and the Environment, University of Southern Queensland, Toowoomba, QLD 4350 Australia; 4 UK Centre for Ecology & Hydrology, Penicuik, Midlothian EH26 0QB UK; 5 Department of Civil, Architectural and Environmental Engineering, University of Texas at Austin, Austin, TX USA; 6 School of Geography, Earth and Environmental Sciences, University of Birmingham, Edgbaston, Birmingham, B15 2TT UK

**Keywords:** Ozonolysis, Biogenic Volatile Organic Compounds, Reaction products, Gas phase, Gas Chromatography Mass Spectrometry, Proton Transfer Reaction Mass Spectrometry

## Abstract

Biogenic volatile organic compounds (bVOCs), synthesised by plants, are important mediators of ecological interactions that can also undergo a series of reactions in the atmosphere. Ground-level ozone is a secondary pollutant generated through sunlight-driven reactions between nitrogen oxides (NO_x_) and VOCs. Its levels have increased since the industrial revolution and reactions involving ozone drive many chemical processes in the troposphere. While ozone precursors often originate in urban areas, winds may carry these hundreds of kilometres, causing ozone formation to also occur in less populated rural regions. Under elevated ozone conditions, ozonolysis of bVOCs can result in quantitative and qualitative changes in the gas phase, reducing the concentrations of certain bVOCs and resulting in the formation of other compounds. Such changes can result in disruption of bVOC-mediated behavioural or ecological interactions. Through a series of gas-phase experiments using Gas Chromatography Mass Spectrometry (GC-MS) and Proton Transfer Reaction Mass Spectrometry (PTR-MS), we investigated the products and their yields from the ozonolysis of a range of ubiquitous bVOCs, which were selected because of their importance in mediating ecological interactions such as pollinator and natural enemy attraction and plant-to-plant communication, namely: (*E*)-β-ocimene, isomers of α and β-farnesene, α-terpinene and 6-methyl-5-hepten-2-one. New products from the ozonolysis of these compounds were identified, and the formation of these compounds is consistent with terpene-ozone oxidation mechanisms. We present the degradation mechanism of our model bVOCs and identify their reaction products. We discuss the potential ecological implications of the degradation of each bVOC and of the formation of reaction products.

**Supplementary Information:**

The online version contains supplementary material available at 10.1007/s10886-023-01467-6.

## Introduction

 Biogenic volatile organic compounds (bVOCs) are a distinct group of secondary metabolites, synthesized and emitted by plants, with various functional roles in plant ecology (Loreto et al. 2014; Pierik et al. 2014). Globally, the biogenic fraction of total VOC emissions outweighs other anthropogenic VOC sources by as much as an order of magnitude (Guenther et al. 1995; (Böge et al. 2013). Mostly lipophilic and marked by high vapour pressure, bVOCs are a diverse group of compounds broadly categorized as terpenoids, benzenoids, phenylpropanoids, fatty acid derivatives and nitrogen and sulphur containing compounds (Dudareva et al. 2004, 2013). Ecologically, bVOCs can function as plant defence compounds against biotic and abiotic stressors and can mediate plant interactions with other organisms across trophic levels (Holopainen 2004; Loreto et al. 2014; Pierik et al. 2014). Terpenoids constitute the largest and most diverse group of bVOCs and follow a basic biosynthesis pathway - the formation of basic C_5_ (hemiterpene) units, which further condense in groups of two or three to form C_10_ (monoterpenes), C_15_ (sesquiterpenes) and C_20_ (diterpenes) products, most of which have high enough vapour pressures to be released into the air from plant tissues under normal atmospheric conditions (Dudareva et al. 2004).

Terpenoids are one of the most important bVOC groups with functional roles in ecology (Vickers et al. 2009) and atmospheric chemistry (Fehsenfeld et al.^1992^), they can be induced and emitted in higher quantities as plant defence responses under a range of abiotic and oxidative stresses (Loreto and Schnitzler 2010). Plants may also emit terpenes in response to herbivore feeding and/or oviposition to deter herbivory or to attract herbivore natural enemies (Büchel et al. 2011; Boncan et al. 2020). Common terpenoids like the monoterpene (*E*)-β-ocimene and the sesquiterpene (*E,E*)*-*α-farnesene have been confirmed to attract herbivore natural enemies through behavioural studies (Dicke et al. 1990; Agrawal et al. 2002). Terpenes also form part of floral bVOC bouquets used as foraging cues for beneficial insects like pollinators (Schiestl 2010). Upon release into the atmosphere, terpenes can act as precursors in the formation of tropospheric ozone (O_3_) and secondary organic aerosol (SOA) particles, these oxidation reactions may undermine their ecological functions especially in polluted atmospheres (Pinto et al. 2010; Blande et al. 2014). Due to their structural characteristics, being mostly unsaturated hydrocarbons with one or more carbon-carbon double bonds, terpenes are classified among the most reactive compounds found in the atmosphere, with lifetimes that range from minutes to hours (Atkinson and Arey 2003). Consequently, they are highly reactive with atmospheric constituents such as O_3_, nitrate radicals (NO_3_) at night, hydroxyl radicals (OH) during the day, and chlorine radicals (Cl) at coastal sites, leading to the disruption of their ecological function (McFrederick et al. 2009; Blande et al. 2014; Masoud and Ruiz 2021; Ryalls et al. 2022a, b, c) and the formation of various oxidation products (Laaksonen et al. 2008).

The ozonolysis of terpenes proceeds via a complex series of reactions and intermediates. The ozonolysis reaction initiates by a concerted cycloaddition of O_3_ to the various carbon-carbon double bonds of terpene to form cyclic 1, 2, 3-trioxolane intermediates where a three-oxygen atom bridge is inserted while a sigma bond remains between the two carbon atoms (Criegee 1975). These highly unstable species, commonly named primary ozonides (POZ), decompose rapidly to form carbonyl molecules (aldehydes or ketones) and oxide reactive intermediates, referred to as ‘Criegee Intermediates’ (CI), by the cleavage of the remaining carbon-carbon and single oxygen-oxygen bonds (Criegee 1975; Aschmann and Atkinson 1994; Atkinson 2000). The carbonyl oxide has ample internal energy to prompt unimolecular reactions or collisional stabilization. The carbonyl oxide, either undergoes a ring closure to dioxirane or hydrogen migration to a hydroperoxide intermediate. The hydroperoxide subsequently undergoes isomerization or decomposition, which leads to formation of OH, carbonyls, carbon dioxide, and a variety of other products, some of which are key precursors for SOA (Aschmann and Atkinson 1994).

Terpenes are known to contribute to the tropospheric formation of O_3_ via interactions with nitric oxide, and to SOA formation as a result of the gas-to-particle conversion of their oxidation products (Ma et al. 2009). Biotic and abiotic stressors on plants cause an increase in bVOC production and SOA yields (Mentel et al. 2013; Joutsensaari et al. 2015; Yli-Pirila et al. 2016). SOA represent an important component of atmospheric fine particulate matter that has potential impacts on the climate, air quality, human health and plant ecology (Camredon et al. 2010). Vegetation surfaces represent a significant sink for VOCs and their SOA products, and the effects of these depositions on plant ecology are just beginning to be explored (Holopainen et al. 2017; Mofikoya et al. 2020). Particulate matter has received significant attention over many years e.g., because blue haze formation occurs due to terpene oxidation in forested regions (Went 1960). In particular, the generation of SOA from the oxidation of terpenes has been studied intensively in order to identify and quantify oxidation products and to understand reaction mechanisms, which are crucial for atmospheric SOA formation (Holopainen et al. 2017). Research has also been conducted to understand their potential ecological impacts e.g., through their deposition on plant surfaces changing herbivore responses (Li et al. 2016). Therefore, a clear understanding of the reaction of O_3_ with terpenes commonly emitted by plants is vital to establish the potential impacts of air pollution on critical ecological processes.

The objective of the current study was to investigate the ozonolysis of specific ubiquitous bVOCs under O_3_ pollution scenarios that commonly occur in nature, including O_3_ excess, VOC excess, and equal concentrations of O_3_ and VOC. Ozone concentrations in nature can vary significantly, with background concentrations in the Northern hemisphere ranging from 25 to 50 ppb, increasing at a rate of 0.2 -2% per year (Vingarzan 2004), but in localised high pollution episodes concentrations in excess of 200 ppb have been recorded (Air Quality Expert Group 2021). Within the proximity of plant vegetation or a flower from which VOCs are released, VOC concentrations are commonly greater than O_3_. As the VOC plume moves away from the plant or flower, VOC concentration will be diluted in the wider body of air and therefore there will be a point at which the ratio of VOC to O_3_ will approach 1:1, after which further dilution will result in O_3_ excess. We tested the hypothesis that ozonolysis of the four model VOCs selected for this study will result in the loss of the primary VOC and the formation of ecologically/behaviourally active reaction products at ecologically relevant timescales. Through a series of gas-phase experiments using Gas Chromatography Mass Spectrometry (GC-MS) and Proton Transfer Reaction Mass Spectrometry (PTR-MS), we investigated the products and their formation over time from the ozonolysis of (*E*)-β-ocimene, isomers of α and β-farnesene, α-terpinene and 6-methyl-5-hepten-2-one. These VOCs were selected because of their ubiquity and importance in ecological interactions such as attraction of pollinators and natural enemies, and for plant-to-plant communication (Dicke et al. 1990; Agrawal et al. 2002; Knudsen et al. 2006).

## Methods and Materials

### Chemicals and Materials

The compounds studied and their structures are presented in Table 1. (*E*)-β-ocimene was purchased from Bedoukian Research Inc. (95.7% (*E*)-β-ocimene and 2.8% (*Z*)-β-ocimene). Isomers of α and β-farnesene, α-terpinene (≥ 95% purity) and 6-methyl-5-hepten-2-one (99% purity) were purchased from Sigma-Aldrich®/Merck at high purity. Compounds were degassed before use by repeated freeze-pump-thaw cycles.

**Table 1:**
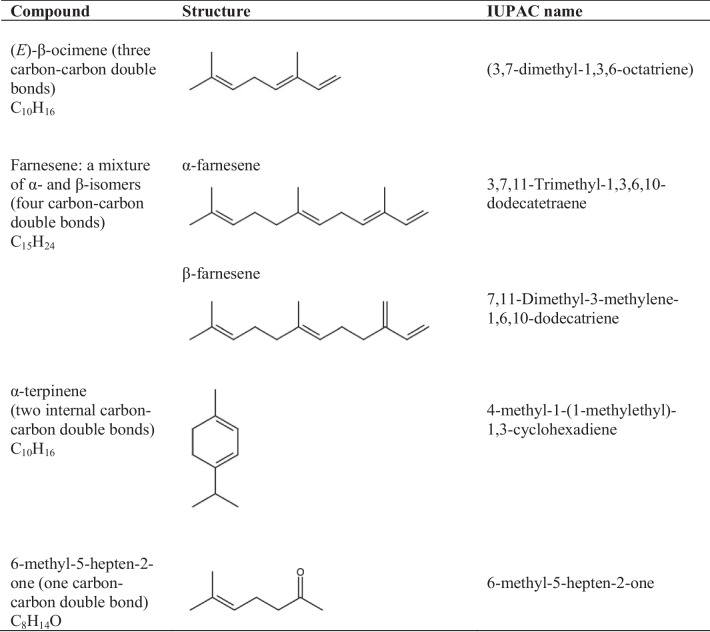
Structures and synonyms of the volatile organic compounds used in this study for the identification of gas phase oxidation products

### SPME Fibres

Gas phase ozonolysis products of the study compounds were collected for analysis on solid phase microextraction (SPME) fibres. A fused-silica fibre coated with 50/30 µm coating Divinylbenzene Carboxen Polydimethylsiloxane (DVB-CX PDMS) (Supelco) was the most efficient fibre among others we tested (PDMS and polyacrylate) during preliminary trials, due to the efficient adsorption to its multilayer coating of the oxygenated products obtained. Before use the fibres were conditioned in a GC inlet according to the manufacturer’s instructions.

### Ozone and Sample Preparation

Ozone was generated as a mixture in oxygen (O_2_) by passing O_2_ through an A2Z 20G Lab Model O_3_ generator (A2Z Ozone Inc, Louisville, Kentucky, USA), and its purity was determined by UV spectroscopy at λ = 254 nm using a dedicated cell with CaF_2_ windows (Pike Technologies). For each experiment a mixture of the VOC under study and cyclohexane as an OH radical scavenger was prepared in an 80 L collapsible Teflon chamber (Adtech Polymer Engineering Ltd) using dry synthetic air (N_2_ + 20 ± 2% O_2_, Air Liquide) as a diluent gas (see Stewart et al. 2013 for procedure details). Typical hydrocarbon concentrations employed were 4.9 × 10^12^ molecule cm^−3^. The VOC and O_3_ concentrations for each experiment are detailed in Supplementary Tables S1-S4. A range of values were investigated to allow us to identify as wide a range of potential products as possible from the studies, therefore we conducted experiments where O_3_ was in excess, where VOC was in excess and where O_3_ and VOC were introduced in a 1:1 ratio. Cyclohexane concentrations were determined using the rate coefficients of the terpene and the scavenger reaction with OH radicals, so that > 95% of OH radicals were scavenged. Experiments were conducted in the absence of cyclohexane because during the GC-MS analyses (see below), cyclohexane dominated the chromatograms at shorter retention times (ca. 2.2 min), making identification of faster eluting compounds challenging. While the reported rate coefficients (e.g. Smith et al. 1996; Kim et al. 2011) differ by ca. 5 to 6 orders of magnitude when comparing the VOCs’ reactivity towards O_3_ and OH, we found no evidence of interference of OH radicals when comparing experimental runs with and without scavenger. We demonstrated, for example by conducting seven experimental runs using the scavenger for 6-methyl-5-hepten-2-one (Table S4), that the results with and without scavenger were consistent. Prior to each experiment, the chamber was thoroughly cleaned by purging with synthetic air. Experiments were carried out by admitting a known concentration of O_3_ into a 1 L Pyrex reaction chamber and adding a sample of the hydrocarbon mixture from the Teflon bag such that a total pressure of 1 atmosphere (760 ± 10 Torr) was achieved. Experiments were carried out at 298 ± 2 ^o^K. Sufficient time (ca. 60 min) was allowed for reactions to take place and for products to be adsorbed on a solid phase microextraction (SPME) fibre. An SPME syringe needle was inserted into the reaction chamber through a SUPELCO Thermogreen™ septum (7/16”); the plunger was depressed to allow the VOC products adsorption onto the fibre coating.

### GC-MS Analysis

Gas Chromatography-Mass Spectrometry was used to facilitate identification of the products generated in the ozonolysis studies. Product analysis was carried out using a Thermo Scientific Finnigan Trace GC-MS operated in EI mode (70 eV) by injecting the SPME fibre into the inlet of the GC. No pre-concentration or pre-treatment of the samples were necessary (Lee et al. 2006). We selected not to conduct chemical derivatisation of the ozonolysis products because this approach is known to induce artefacts and peak identification uncertainties (Jaoui et al. 2003). The oven temperature was initially held at 40 °C then increased linearly to 200 °C at 10 °C / min. The GC inlet was kept at 220 °C and the gas chromatograph/mass spectrometer interface temperature was 250 °C. Compound separation was carried out on an RTx-5MS capillary column (30 m, 0.25 mm ID, 0.25 μm, Restek Thames). High purity helium (99.995%, Air Liquide) was used as a carrier gas. Masses were scanned in the 25 to 250 amu range. At least eight chromatograms were collected for each VOC (Tables S1-S4). Only peaks that appeared consistently, were symmetrical, well resolved and with signal-to-noise ratios clearly above those of blank runs, were taken into consideration when identifying terpene oxidation products. Most VOC degradation products were consistently observed across all experimental data sets and showed very similar fragmentation patterns under most experimental conditions.

### PTR-MS Analysis

To investigate the timescales over which degradation of gas-phase parent terpenes and formation of their oxidation products occurred, additional measurements were made using PTR-MS (Ionicon Analytik GmbH, Austria). Air was sampled from a collapsible Teflon chamber (Adtech Polymer Engineering Ltd) using Teflon tubing and pulled through a 0.45 μm pore size Whatman PTFE particulate filter before entering the PTR-MS. Dry synthetic air (Air Liquide) was used as a diluent gas in these measurements. The PTR-MS was a quadrupole mass spectrometer that uses hydronium ions (H_3_O^+^) to chemically ionize the compound of interest through a proton transfer reaction. Thus, compounds with a proton affinity higher than that of water can be detected by the PTR-MS and were identified by their mass to charge ratios (*m/z*). However, compounds with the same molecular weight cannot be distinguished by PTR-MS, since this method identifies compounds alone by their molecular weight plus one (H^+^) and operates at unit mass resolution. The increase of count rates for certain ions indicates the formation of oxidation products. Possible oxidation products are presented in this work based on the oxidation mechanisms of the parent terpene. PTR-MS data were collected at drift tube voltages of 500 V, 550 and 600 V which corresponded to *E/N* ratios of 122 Td, 134 Td and 147 Td (1 Townsend, Td = 10^−17^cm^2^V molecule^−1^), respectively. Where, *E* is the electric field and *N* is the number density of molecules in the drift tube.

### Oxidation Product Identification

Oxidation compounds for which a standard was commercially available were confirmed by comparison of the mass spectra of the compound with the authentic spectra run in the same experimental conditions (acetaldehyde, acrylic acid, 3-methylfuran-2,5-dione and 6-methyl-5-hepten-2-one); retention time was used as an additional confirmation of the product. All compounds recorded on blank SPME fibres and background peaks were eliminated prior to identification of product peaks. Only mass spectra of symmetrical and well resolved GC peaks were taken into consideration when identifying terpene oxidation products. In some cases, it was not possible to associate some spectral peaks with a compound or a structure due to the complexity of assigning structure/functional groups to those mass spectra and the lack of authentic standards.

## Results and discussion

**Scheme 1:**
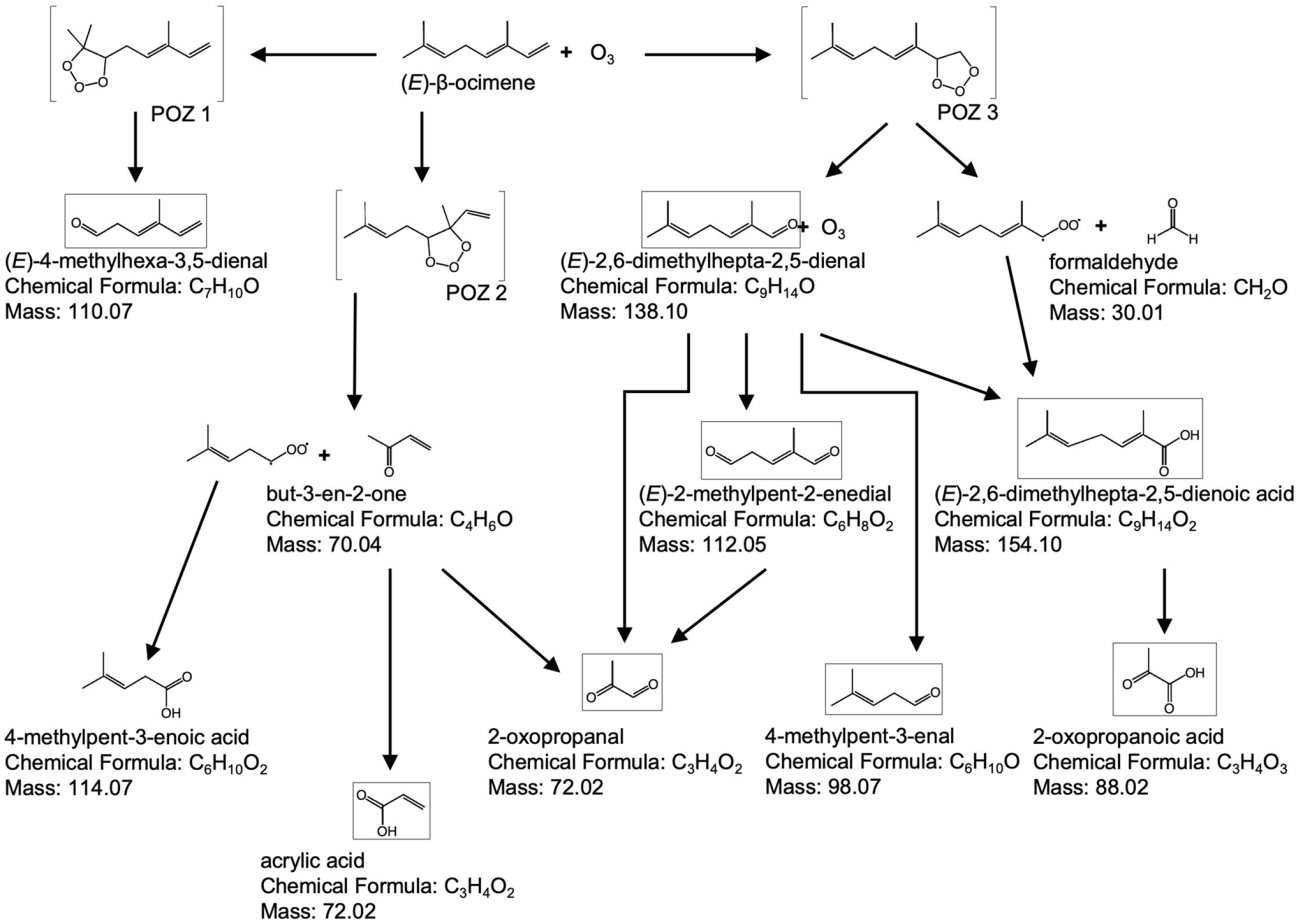
Simplified mechanism of the ozonolysis of (*E*)-β-ocimene illustrating those products identified during the current study in square boxes. The primary ozonides (POZ) in square brackets are those previously by Jenkin et al. 2020

New products from the reaction of O_3_ with (*E*)-β-ocimene, isomers of α and β-farnesene, α-terpinene and 6-methyl-5-hepten-2-one were identified. The main oxidation products are summarized in Table 2. The compounds identified in this work are consistent with the terpene-O_3_ oxidation mechanisms (see Scheme 1 for ozonolysis mechanisms for (*E*)-β-ocimene; for a partial mechanism for α-terpinene see Lee et al. 2006; for a detailed ozonolysis mechanism of α and β-farnesene see Jaoui et al. 2017; for 6-methyl-5-hepten-2-one a full ozonolysis mechanism has not yet been proposed to our knowledge beyond that described by Leonardo et al. 2008, which was limited to computational methods).

**Table 2:** Oxidation products detected from the ozonolysis of (*E*)-β-ocimene, isomers of α and β-farnesene, α-terpinene and 6-methyl-5-hepten-2-one from GC-MS analyses. Reaction products were identified by their spectra (unless otherwise stated). Those studies which, to the authors knowledge, have previously identified and named these gas phase reaction products as occurring from the ozonolysis of the volatile organic compound under investigation are identified in the final column. In addition to the products identified, the *m/z *of each volatile organic compound’s predicted ozonolysis products, based on general reaction mechanism, are stated for comparison

Volatile Organic Compound	Oxidation product			
	*m/z*	Chemical Formula	Nomenclature	Previously identified as a gas phase reaction product
(*E*)-β-Ocimene	72	C_3_H_4_O_2_	2-oxopropanal/acrylic acid*	Calogirou et al. 1999
	88	C_3_H_4_O_3 _	2-oxopropanoic acid	N/A
	98	C_6_H_10_O	4-methylpent-3-enal	N/A
	110	C_7_H_10_O	(*E*)-4-methylhexa-3,5-dienal	Reissell et al. 2002
	112	C_6_H_8_O_2_	(*E*)-2-methylpent-2-enedial	N/A
	138	C_9_H_14_O	(*E*)-2,6-dimethylhepta-2,5-dienal	N/A
	154	C_9_H_14_O_2_	(*E*)-2,6-dimethylhepta-2,5-dienoic acid	N/A
	30, 58, 70, 72, 88, 98,102, 110, 112,114, 138		Products predicted by applying general reaction mechanisms for ozonolysis	
Isomers of α and β-farnesene	72	C_3_H_4_O_2_	2-oxopropanal	Kourtchev et al. 2009
	100	C_5_H_8_O_2_	4-oxopentanal	Kourtchev et al. 2009
	110	C_7_H_10_O	4-methylhexa-2,4-dienal	N/A
	162	C_7_H_14_O_4_	4,5-Dihydroxy-5-methyl-hexanoic acid	N/A
	206	C_14_H_22_O	(*E*)-6-10-dimethyl-2-methyleneundeca-5,9-dienal	Jaoui et al. 2017
	222	C_14_H_22_O_2_	(3*E*,5*E*)-2,6,10-trimethylundeca-3,5,9-trienoic acid	N/A
	44, 46, 58, 60 72, 74, 84, 88, 98, 100, 110, 114, 116, 126, 128, 130, 144,152, 156, 178, 180, 182, 192, 196, 206, 210, 212, 222		Products predicted by applying general reaction mechanisms for ozonolysis	
α-terpinene	112	C_5_H_4_O_3_	3-methylfuran-2,5-dione*	N/A
	124	C_8_H_12_O	6-methyl-3,5-heptadien-2-one	N/A
	126	C_8_H_14_O	6-methyl-5-hepten-2-one*	N/A
	142	C_8_H_14_O_2_	6-methylheptane-2,5-dione	N/A
	156	C_9_H_16_O_2_	2-Hydroxy-2,6-dimethyl-hept-6-en-3-one	N/A
	168	C_10_H_16_O_2_	3,7-Dimethyl-6-oxo-2-octenal	N/A
	44, 46, 60, 62, 74, 112, 148, 156, 172, 184, 186, 200		Products predicted by applying general reaction mechanisms for ozonolysis	
6-methyl-5-hepten-2-one	72	C_3_H_4_O_2_	2-oxopropanal	N/A
	100	C_5_H_8_O_2_	4-oxopentanal	Colmán et al. 2015
	58, 60, 74, 100, 116		Products predicted by applying general reaction mechanisms for ozonolysis	

* products identified by injection of a commercial standard into the GC-MS under identical conditions

### (E)-β-Ocimene Oxidation Products

Scheme 1 displays a simplified mechanism for the degradation of (*E*)-β-ocimene illustrating the major products we identified in square boxes. An oxidation product with a molecular weight of 110 was observed from OH and O_3_ reactions with (*E*)-β-ocimene (Table 2, Fig. S1). The fragmentation pattern showed characteristic losses of CH_3_, CO and HCO from the molecular ion. This indicates the formation of a C_7_ aldehyde from the cleavage of a C_3_H_6_ radical from (*E*)-β-ocimene to form 4-methylhexa-3,5-dienal (see primary ozonide (POZ) 1 channel to the left in Scheme 1). This is consistent with the previously reported formation of this compound (Calogirou et al. 1999; Reissell et al. 2002; Baker et al. 2004); recent work has estimated that POZ 1 is the main channel contributing ca. 85% (Jenkin et al. 2020).

While only a minor channel according to Jenkin et al. (2020) and contributing less than 5%, we found evidence of POZ 2 formation: the cleavage of the double bond on C_3_ of (*E*)-β-ocimene leads to formation of POZ 2 which rapidly decomposes to produce 4-methylpent-3-enoic acid (114 *m/z*) and but-3-en-2-one (see central channel in Scheme 1; consistent with Guanouni et al. (2020). Isomers of mass 72, acrylic acid and 2-oxopropanal (Fig. S2) may form from an intermediate radical (3-peroxybut-1-ene) and but-3-en-2-one, by delocalization of oxygen from the peroxy radical and the formation of a carbonyl group.

**Fig. 1:**
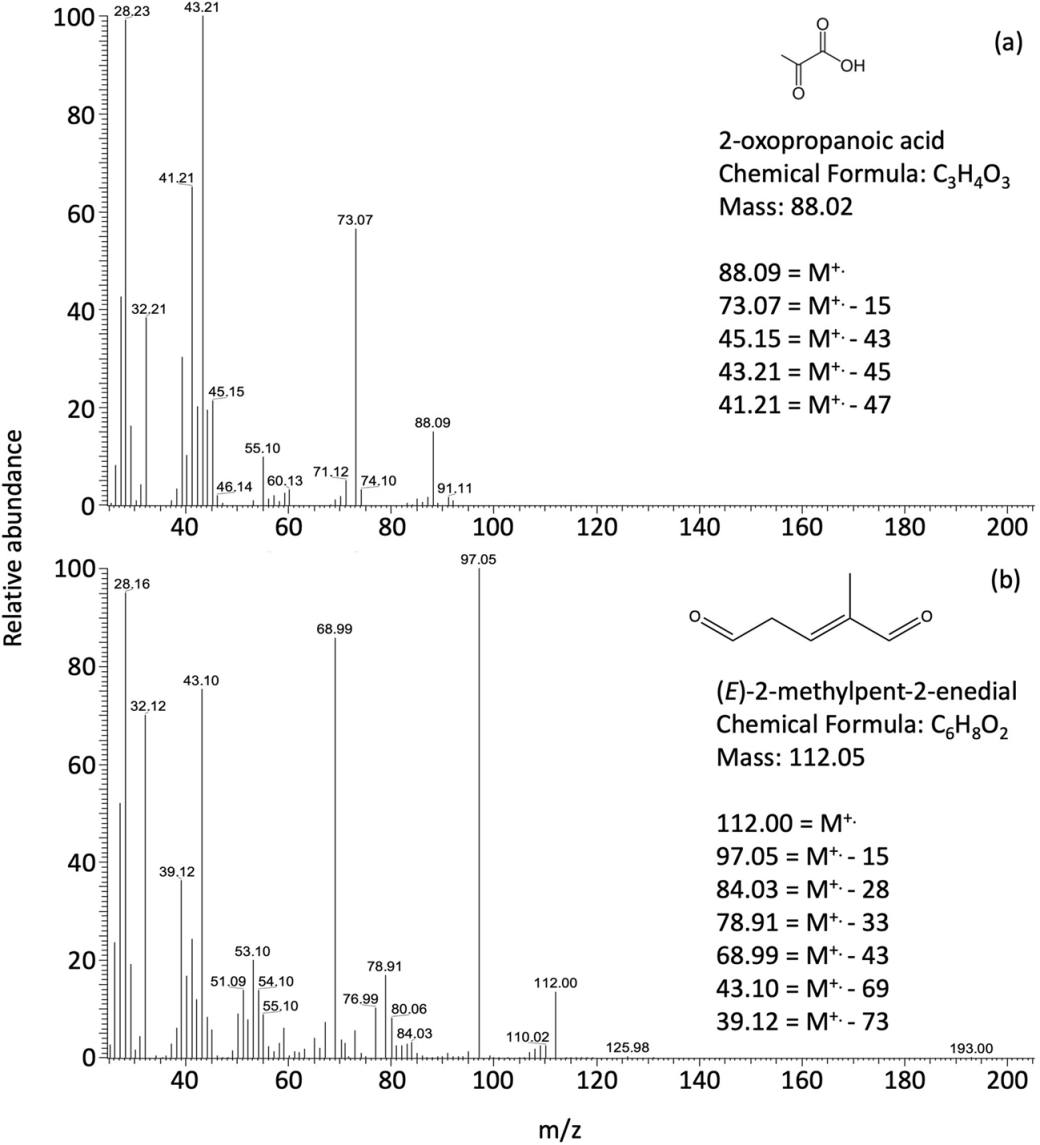
Representative mass spectra of (*E*)-β-ocimene ozonolysis products (**a**) mass 88, identified as 3-oxopropanoic acid, (**b**) mass 112, identified as: (*E*)-2-methylpent-2-enedial. M^+.^ indicates the molecular ion

The cleavage of the C_1_ double bond of (*E*)-β-ocimene, forming POZ 3, leads to the formation of C_7_ carboxylic acids (contributing ca. 10% to the overall ozonolysis according to Jenkin et al. 2020), (*E*)-2,6-dimethylhepta-2,5-dienal of mass 138 and (*E*)-2,6-dimethylhepta-2,5-dienoic acid of mass 154. The formation of mass 154 (Scheme 1) has an energy barrier of 97.82 kcal mol^−1^ (Sun et al. 2011). The identification of the acid was based on the mass 154 fragmentation that shows a loss of M-17 and M-45 indicating the formation of a short-chain acid. Subsequently, the carbon-carbon double bond on the C_2_ of the acid may be attacked by O_3_ to form a carbonyl group resulting in a lower molecular weight acid, 2-oxopropanoic acid (88 *m/z*). Identification was based on the fragmentation spectra, which indicated the loss of CH_3_ M-15, and the loss of COOH M-45 (Fig. 1a). The oxidation product of mass 138 ((*E*)-2,6-dimethylhepta-2,5-dienal) may cleave the double bond at C_5_ to form a carbonyl resulting in (*E*)-2-methylpent-2-enedial (112 *m/z*). The mass spectrum (Fig. 1b) shows the fragmentation of the aldehyde with mass losses of M-15, M-28 and M-43 corresponding to CH_3_, CO and CH_2_-CH-O; this is indicative of the aldehyde formation with an ion mass of 112. The two products identified at 72 and 98 *m/z* (Figs. S2 and S3) can also form from the oxidation of (*E*)-4-methylhexa-3,5-dienal with OH and O_3_. The reaction rates of (*E*)-4-methylhexa-3,5-dienal with OH radical and O_3_ were estimated to be 1.61 ± 0.35 × 10^−10^ and 4.13 ± 0.81 × 10^−17^ cm^3^ molecule^−1^ s^−1^, respectively (Baker et al. 2004). The formation of a C_6_ aldehyde (*m/z* 98) may result from the breakdown of the endo/internal carbon-carbon double bond in (*E*)-β-ocimene. The fragmentation pattern of this aldehyde (Fig. S3) shows mass losses of M-18 (loss of water molecule), M-28 (loss of ethylene), M-29 (cleavage of COH^+^), M-43 (loss of CH_2_-CH-O) and M-44 (loss of CH_2_ = CH-OH). This product is identified as 4-methylpent-3-enal. The formation energies for C_3_H_4_O_2_ (*m/z* 72), C_6_H_10_O (*m/z* 98), C_9_H_14_O (*m/z* 138), C_6_H_8_O_2_ (*m/z* 112) and C_9_H_14_O_2_ (*m/z* 154) were estimated by density functional theory calculations (Sun et al. 2011). Formation of mass 138 has an energy barrier of only 26.97 kcal mol^−1^, while the formations of masses 98, 72 and 112 have potential barriers of 45.99, 50.77 and 51.92 kcal mol^−1^, respectively (Sun et al. 2011).

Other masses/molecular ions were also consistently observed from the GC-MS spectra, but due to the lack of commercial standards and the complexity of the mass spectra it was not possible to identify those products.

**Fig. 2:**
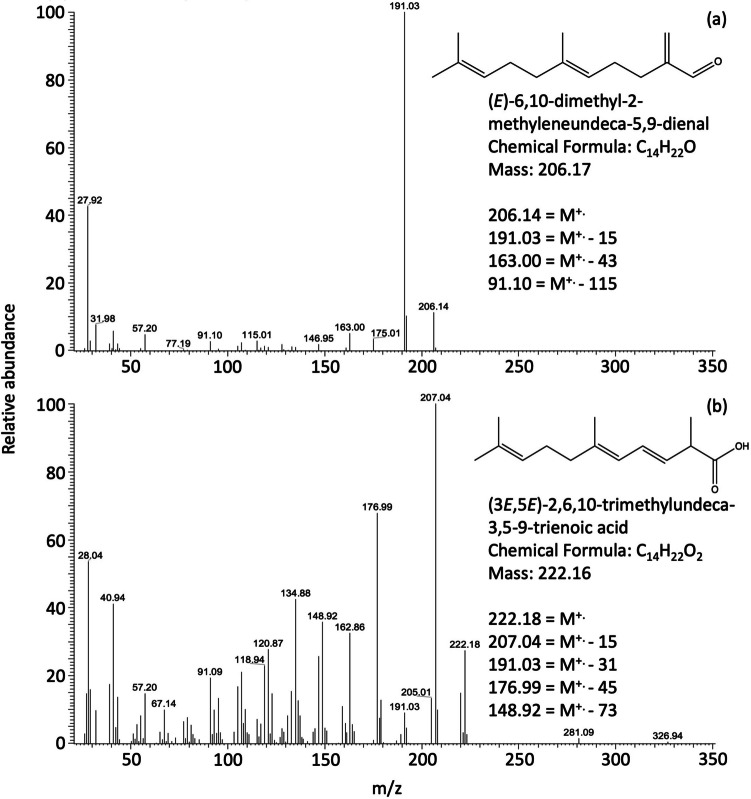
Representative mass spectra of isomers of α and β-farnesene ozonolysis products (**a**) mass 206, identified as: (*E*)-6,10-dimethyl-2-methyleneundeca-5,9-dienal, (**b**) mass 222, identified as: (3*E*,5*E*)-2,6,10-trimethylundeca-3,5,9-trienoic acid. M^+.^ indicates the molecular ion

### Isomers of α and β-Farnesene Oxidation Products

Gas-phase isomers of α and β-farnesene oxidation products are listed in Table 2. Low molecular weight products identified in this work are consistent with those reported by Jaoui et al. (2017). 2-oxopropanal (*m/z* 72) and a product of *m/z* 162 was identified as 4,5-dihydroxy-5-methyl-hexanoic acid (Jaoui et al. 2017). The spectra in the Supplementary information (Fig. S4) shows the fragmentation patterns that support the formation of this acid. An oxidation product of *m/z* 100 shows mass losses of M-29 and M-45. A product with a molecular weight of 206 was also observed from the ozonolysis (Fig. 2a). 4-methylhexa-2,4-dienal (*m/z* 110) was identified: its fragmentation pattern is consistent with the loss of masses 28 and 43. This product is formed from the degradation of α-farnesene; for a detailed ozonolysis mechanism for α and β-farnesene see Scheme in Jaoui et al. (2017). A product of mass 222 has a fragmentation pattern supporting the formation of an acid with mass losses of 15 and 45 (Fig. 2b).

**Fig. 3:**
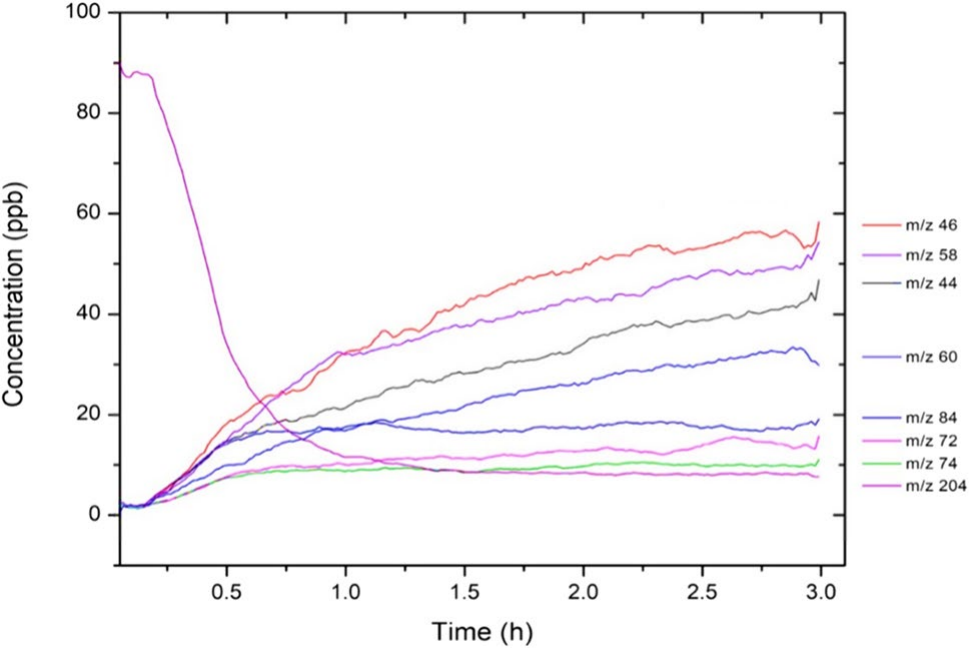
Time profiles of ions detected using PTR-MS during the ozonolysis of isomers of α and β-farnesene. The eight ions displayed include the molecular ion of α and β-farnesene (*m/z* = 204), and the molecular ions of products identified in the GC-MS analysis or predicted to be formed by the ozonolysis of α and β-farnesene by applying general reaction mechanisms. Note that PTR-MS measurements show the unprotonated mass-to-charge ratios

Isomers of α and β-farnesene oxidise within approx. 30 min of the ozonolysis (see Fig. 3 for the time profile of the reaction determined by PTR-MS), forming a number of low molecular weight product ions at low concentrations. Kim et al. (2011) calculated the ozonolysis rate coefficients for α- and β-farnesene to be 10.4 × 10^−16^ and 8.74 × 10^−16^ cm^3^ molecule^−1^ s^−1^, respectively. The rapid ozonolysis of α and β-farnesene we confirmed here may have significant ecological implications such as O_3_ quenching in plants as detailed later.

### α-Terpinene Oxidation Products

**Fig. 4:**
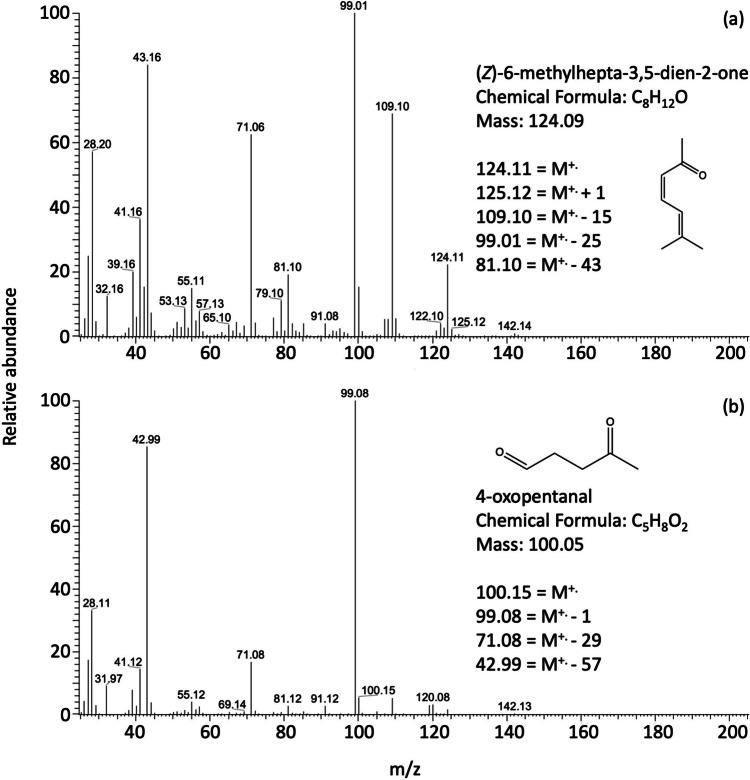
Representative mass spectra of α-terpinene ozonolysis products (**a**) mass 124, identified as: 6-methylhepta-3,5-dien-2-one, (**b**) mass 100, identified as: 4-oxopentanal. M^+.^ indicates the molecular ion

Seven major gas-phase α-terpinene oxidation products were observed in our experiments (Table 2 and Fig. S5). The oxidation of α-terpinene is more complicated than both (*E*)-β-ocimene, and α and β-farnesene. α-terpinene contains a ring structure with two endo double bonds. For the ozonolysis of α-terpinene high yields of first generation products are expected with low yields of second generation products (Lee et al. 2006). Lee et al. (2006) observed no high product yields except at *m/z* 138 which decreased by 50% in two hours. They proposed a partial mechanism including a product observed with a molecular ion of 142 *m/z*; several pathways were proposed for the formation of this product (Lee et al. 2006) consistent with the high yield of 6-methylheptane-2,5-dione (*m/z* 142) that we observed. Other compounds we observed have masses of 126 *m/z* and 124 *m/z* and are identified as 6-methyl-5-hepten-2-one (C_8_H_14_O) and 6-methyl-3,5-heptadien-2-one (C_8_H_12_O), respectively. 6-methyl-5-hepten-2-one eluted at the same time as the commercial standard and their spectra were consistent with each other, which directly confirms the formation of this ketone. The fragmentation pattern for the product with *m/z* 124 shows peaks at 81, 99, 109 and 125 corresponding to mass losses of M-43, M-25, M-15 and M+1 (see Fig. 4a). The observation of 6-methylhepta-3,5-dien-2-one (C_8_H_12_O, *m/z* 124) is difficult to explain in terms of the currently assumed α-terpinene ozonolysis reaction mechanism (compare e.g. Scheme in Lee et al. 2006).

The ozonolysis products of α-terpinene include *m/z* 156, 112 and 168; these were stable products and identified as 2-Hydroxy-2,6-dimethyl-hept-6-en-3-one (C_9_H_16_O_2_), 3-methylfuran-2,5-dione (C_5_H_4_O_3_) and 3,7-Dimethyl-6-oxo-2-octenal (C_10_H_16_O_4_), respectively (Table 2). The 3-methylfuran-2,5-dione (*m/z* 112) mass spectrum was compared with the commercial standard spectrum: the two spectra had very similar fragmentation patterns and eluted at the same time, directly confirming the identity of this product. The products of masses 112, 142, 156 and 168 formed are consistent with Lee et al. 2006.

### 6-Methyl-5-Hepten-2-One Oxidation Products

A compound of mass 72 was observed from the ozonolysis of 6-methyl-5-hepten-2-one; with a loss of M-15 (for a CH_3_ removal), M-29 (for COH+) and M-43 it was identified as 2-oxopropanal. Another oxidation product of mass 100 was detected and identified as 4-oxopentanal (Fig. 4b).

The O_3_ reaction with 6-methyl-5-hepten-2-one follows the Criegee mechanism of alkene ozonolysis described earlier. Previous studies employed density functional theory methods to determine the structure and energies of the reactant, intermediates, transition states and products (Leonardo et al. 2008). There are currently too few identified products to propose a full mechanism for this reaction, but our findings are consistent with the basic scheme proposed by Leonardo et al. (see Scheme in Leonardo et al. 2008).

### Ecological Implications of Ozonolysis

bVOCs are emitted by vegetative and floral plant parts and are important for eliciting herbivore responses across trophic levels (Holopainen 2004; Knudsen et al. 2006). Terpenes as a bVOC group are critical for these interactions and our study emphasises their vulnerability to ozonolysis. The ubiquity of our selected compounds and the various ecological interactions they mediate raises the question of the fragility of those interactions under elevated O_3_ episodes. (*E*)-β-ocimene is involved in multiple interactions between plants and other organisms, especially as a generalist attractant to a wide spectrum of pollinators and in plant-to-plant communication (Farré-Armengol et al. 2017; Arimura et al. 2012). Ozonolysis of (*E*)-β-ocimene can lead to reductions in its atmospheric concentrations (McFrederick et al. 2008) and alter its attractiveness to herbivores and pollinators (Masui et al. 2020; Farré-Armengol et al. 2016). Some of the reaction products formed by the oxidation of (*E*)-β-ocimene identified in this study e.g., 4-methylhexa-3,5-dienal are common volatiles themselves (Reissell et al. 2002). Ozone concentrations are predicted to continue to rise (Vingarzan 2004; IPCC 2013); therefore, the reactions we define may lead to further reductions in the quantity and quality of odour cues available to foraging insects, which may affect the cues recipients, especially specialist insects that may have evolved specific receptors to a biogenic signal (McFrederick et al. 2009).

Those plant-pollinator interactions mediated by sesquiterpenes, including the farnesenes, are thus particularly vulnerable to disruption due to rapid ozonolysis (as confirmed by our PTR-MS results – Fig. 3) and the relatively long distance of the interactions they mediate (McFrederick et al. 2009). The rapid ozonolysis of the farnesenes also make them ideal for O_3_ quenching within plants (they are induced in response to biotic and abiotic stresses), although their overall oxidative stress protection capacity is not clear (Heiden et al. 1999; Palmer-Young et al. 2015). There is some evidence for changes in herbivore responses due to the ozonolysis of sesquiterpenes (Li and Blande 2015; Li et al. 2016), although whether those changes are due to loss of the sesquiterpene compound, or the formation of reaction products remains unclear. However, a recent study by Mofikoya et al. (2020) showed that the deposition of α-pinene oxidation products on cabbage plants reduced herbivore feeding and oviposition.

bVOCs are generally released by plants as a blend of multiple compounds in different ratios with a certain ratio of those compounds often being critical for eliciting herbivore responses (Bruce et al. 2005; Bruce and Pickett 2011). The ozonolysis of reactive compounds within a blend may alter the signal efficacy, particularly over longer distances (Blande et al. 2014; Farré-Armengol et al. 2016). It has been proposed that some oxidized forms of emitted compounds or fragments such as the C_2_-C_6_ fragments of monoterpenes may be of signalling value, along with the original compound, especially in long distance communication (Šimpraga et al. 2016). For example, 6-methyl-5-hepten-2-one (also known as sulcatone), a bVOC widely reported in the literature as being constitutively emitted, was shown to be an ozonolysis product of α-terpinene in the present study. 6-methyl-5-hepten-2-one has previously been shown to be an ozonolysis product of the oxidation of sesquiterpenes on leaf surfaces (Fruekilde et al. 1998; Acton et al. 2018) and of squalene from human skin lipids (Wisthaler and Weschler 2010), and is known to elicit a variety of behavioural responses in a variety of insects, including herbivores and pollinators, acting in different species as a pheromone, an attractant and an allomone (e.g. Duffield et al. 1977; Du et al. 1998; Page et al. 2014). Another reaction product of α-terpinene, 6-methyl-3,5-heptadien-2-one, has previously been identified to be emitted from a number of orchids (Kaiser 1993) and has been identified from female Eastern larch beetles, *Dendroctonus simplex*, with potential function as a pheromone (Francke et al. 1995). However, most of the reaction products identified in this study are, to our knowledge, not currently known to elicit behavioural activity in any insect species, suggesting that for the VOCs in this study the primary mechanism by which ozonolysis is likely to influence insect behaviour is through the VOCs reduction rather than through the generation of alternative behaviourally active VOC reaction products.

Our results demonstrate the vulnerability of terpene-mediated communication to the ongoing increases in global tropospheric O_3_ concentrations. Our findings strongly encourage further research to elucidate the specific impacts of ozonolysis on the chemical ecology of the diverse range of ecological interactions mediated by such compounds, and on the potential wider impacts on insect and plant biodiversity and the ecosystem services these organisms provide.

### Supplementary Information

Below is the link to the electronic supplementary material.

ESM 1 (DOCX 79.5 KB)
