# Supplementary material for: Atmospheric Degradation of Ecologically Important Biogenic Volatiles: Investigating the Ozonolysis of (E)-β-Ocimene, Isomers of α and β-Farnesene, α-Terpinene and 6-Methyl-5-Hepten-2-One, and Their Gas-Phase Products
Source: J Chem Ecol. 2024 Jan 9;50(3-4):129–42. doi: 10.1007/s10886-023-01467-6 (PMC11043181; doi:10.1007/s10886-023-01467-6)
Supplement: Supplementary file 1 — (DOCX 79.5 KB) [file 10886_2023_1467_MOESM1_ESM.docx]

**Supplementary Information**

**Atmospheric degradation of ecologically important biogenic volatiles: Investigating the ozonolysis of (*E*)-β-ocimene, isomers of α and β-farnesene, α-terpinene and 6-methyl-5-hepten-2-one, and their gas phase products.**

Journal of Chemical Ecology

DALILA TOUHAMI, ADEDAYO O. MOFIKOYA, ROBBIE D. GIRLING^*^, BEN LANGFORD, PAWEL K. MISZTAL, AND CHRISTIAN PFRANG^*^

*Corresponding authors: [robbie.girling@unisq.edu.au](mailto:robbie.girling@unisq.edu.au); [c.pfrang@bham.ac.uk](mailto:c.pfrang@bham.ac.uk)

**Table S1** Details of each experiment performed to investigate the ozonolysis of (*E*)-β-ocimene by gas-chromatography mass-spectrometry, defining the (*E*)-β-ocimene and ozone (O_3_) concentrations and whether cyclohexane was used as a hydroxyl radical scavenger

| Run | (E)-β-ocimene conc. (ppm) | (E)-β-ocimene:O_3_ | Cyclohexane |
| --- | --- | --- | --- |
| 1 | 125 | 2:1 | No |
| 2 | 125 | 2:1 | No |
| 3 | 125 | 1:1 | No |
| 4 | 1.18 | 5:1 | No |
| 5 | 0.236 | 1:2 | No |
| 6 | 0.236 | 1:5 | No |
| 7 | 0.460 | 2:1 | No |
| 8 | 0.7 | 3:1 | No |

**Table S2** Details of each experiment performed to investigate the ozonolysis of (*E*)-α and β-farnesene by gas-chromatography mass-spectrometry, defining the (*E*)-α and β-farnesene and ozone (O_3_) concentrations and whether cyclohexane was used as a hydroxyl radical scavenger

| Run | (*E*)-α and β-farnesene conc. (ppm) | (*E*)-α and β-farnesene:O_3_ | Cyclohexane |
| --- | --- | --- | --- |
| 1 | 0.323 | 1:1 | No |
| 2 | 0.323 | 1:1 | No |
| 3 | 0.323 | 1:1 | No |
| 4 | 0.646 | 2:1 | No |
| 5 | 1.61 | 5:1 | No |
| 6 | 0.323 | 1:1 | No |
| 7 | 0.323 | 1:1 | No |
| 8 | 0.323 | 1:1 | No |
| 9 | 0.323 | 1:1 | No |
| 10 | 0.323 | 1:2 | No |
| 11 | 0.646 | 2:1 | No |
| 12 | 1.61 | 5:1 | No |
| 13 | 0.323 | 1:5 | No |
| 14 | 0.323 | 1:1 | No |
| 15 | 0.323 | 1:1 | No |
| 16 | 0.323 | 1:5 | No |
| 17 | 0.646 | 2:1 | No |

**Table S3** Details of each experiment performed to investigate the ozonolysis of α-terpinene by gas-chromatography mass-spectrometry, defining the α-terpinene and ozone (O_3_) concentrations and whether cyclohexane was used as a hydroxyl radical scavenger

| Run | α-terpinene conc. (ppm) | α-terpinene:O_3_ | Cyclohexane |
| --- | --- | --- | --- |
| 1 | 0.601 | 2:1 | No |
| 2 | 1.5 | 5:1 | No |
| 3 | 2.36 | 10:1 | No |
| 4 | 23.6 | 100:1 | No |
| 5 | 300 | 100:1 | No |
| 6 | 300 | 100:1 | No |
| 7 | 300 | 100:1 | No |
| 8 | 250 | 5:1 | No |
| 9 | 250 | 8:1 | No |
| 10 | 250 | 8:1 | No |
| 11 | 130 | 4:1 | No |
| 12 | 150 | 20:1 | No |
| 13 | 150 | 10:1 | No |
| 14 | 150 | 1:1 | Yes |
| 15 | 0.236 | 1:2 | No |
| 16 | 1.5 | 5:1 | No |
| 17 | 14.4 | 10:1 | No |
| 18 | 144 | 100:1 | No |

**Table S4** Details of each experiment performed to investigate the ozonolysis of 6-methyl-5-hepten-2-one by gas-chromatography mass-spectrometry, defining the 6-methyl-5-hepten-2-one and ozone (O_3_) concentrations and whether cyclohexane was used as a hydroxyl radical scavenger

| Run | 6-methyl-5-hepten-2-one conc.  (ppm) | 6-methyl-5-hepten-2-one:O_3_ | Cyclohexane |
| --- | --- | --- | --- |
| 1 | 0.316 | 1:1 | No |
| 2 | 0.316 | 1:2 | No |
| 3 | 0.600 | 2:1 | No |
| 4 | 1.58 | 5:1 | No |
| 5 | 1.58 | 1:5 | No |
| 6 | 0.316 | 1:2 | Yes |
| 7 | 0.316 | 1:2 | No |
| 8 | 0.316 | 1:1 | Yes |
| 9 | 0.316 | 1:1 | No |
| 10 | 0.316 | 1:2 | Yes |
| 11 | 0.600 | 2:1 | Yes |
| 12 | 0.316 | 1:1 | No |
| 13 | 125 | 1:2 | Yes |
| 14 | 0.316 | 5:1 | No |
| 15 | 0.316 | 1:1 | Yes |
| 16 | 0.316 | 1:2 | Yes |

**Fig. S1** Representative mass spectra of (E)-β-ocimene-ozone oxidation product of mass 110, identified as 4-methylhexa-3,5-dienal

**Fig. S2** Representative mass spectra of (*E*)-β-ocimene-ozone oxidation product of mass 72, identified as 2-oxopropanal, 3-oxopropanal or acrylic acid

**Fig. S3** Representative mass spectra of (*E*)-β-ocimene-ozone oxidation product of mass 98, identified as 4-methylpent-3-enal

**Fig. S4** Representative mass spectra of (E)-α and β-farnesene ozone oxidation products of mass 162, identified as 4,5-dihydroxy-5methyl-hexanoic acid


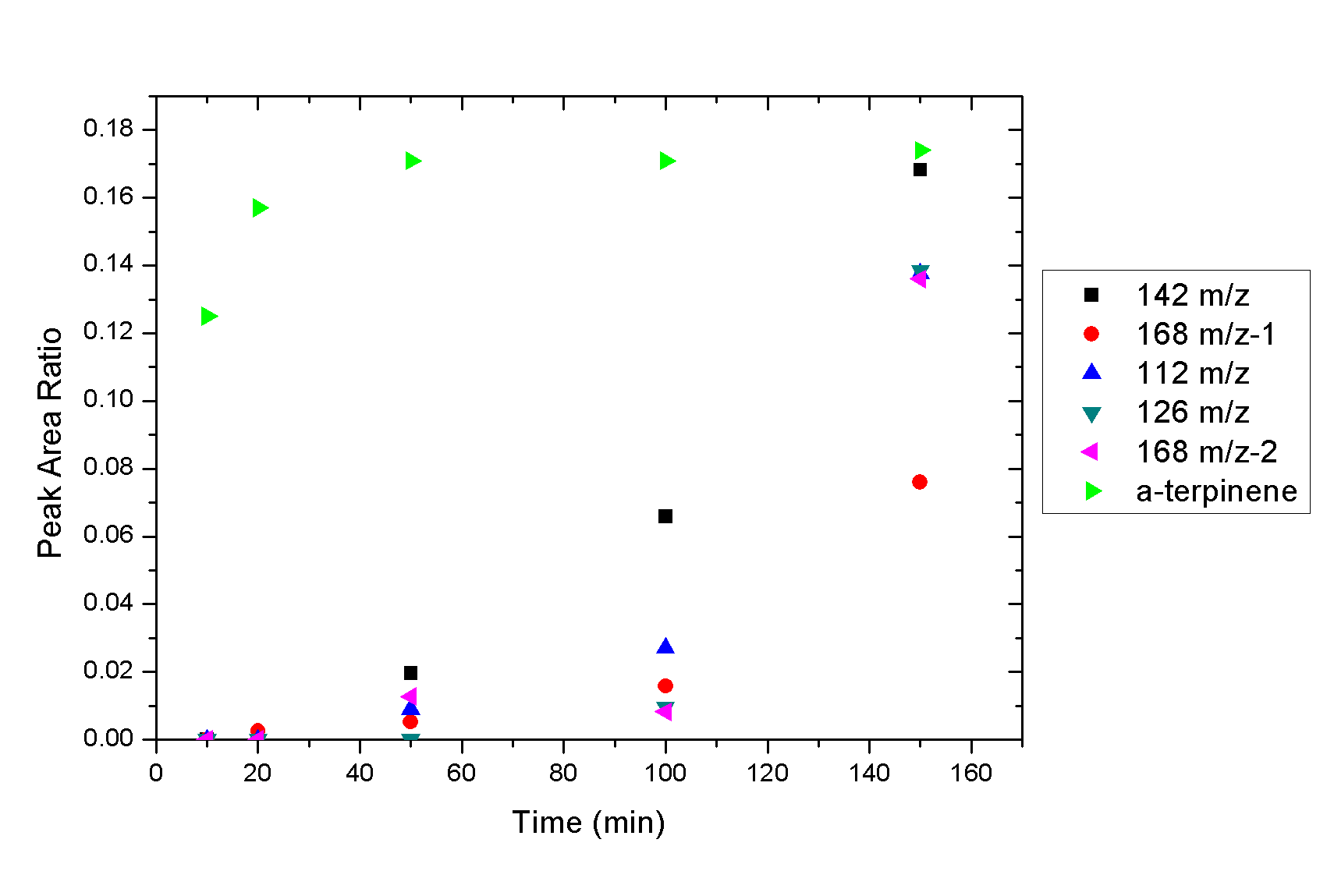


**Fig. S5** Major ion products concentration over time at 2:1 ratio of α-terpinene to ozone detected by SPME GCMS. This figure shows the adsorption of the different ions on the DVB-CX- SPME fibre. Saturation is reached at around 50 min for α-terpinene which allowed determination of the experimental adsorption time for the oxidation products. Yields of the products were not estimated in this work given that not all products adsorb efficiently on the SPME fibre as shown for mass 168 m/z, which was not adsorbed onto the fibre, with only 168 m/z-1 and 168 m/z-2 being adsorbed.
